# Bias Investigation in Artificial Intelligence Systems for Early Detection of Parkinson’s Disease: A Narrative Review

**DOI:** 10.3390/diagnostics12010166

**Published:** 2022-01-11

**Authors:** Sudip Paul, Maheshrao Maindarkar, Sanjay Saxena, Luca Saba, Monika Turk, Manudeep Kalra, Padukode R. Krishnan, Jasjit S. Suri

**Affiliations:** 1 Department of Biomedical Engineering, North Eastern Hill University, Shillong 793022, India; sudip.paul.bhu@gmail.com (S.P.); mahesh.nehu.333@gmail.com (M.M.); 2 Department of CSE, International Institute of Information Technology, Bhuneshwar 751003, India; sanjay@iiit-bh.ac.in; 3 Department of Radiology, University of Cagliari, 09121 Cagliari, Italy; lucasabamd@gmail.com; 4 Department of Neurology, University Medical Centre Maribor, 1262 Maribor, Slovenia; monika.turk84@gmail.com; 5 Department of Radiology, Harvard Medical School, Boston, MA 02115, USA; MKALRA@mgh.harvard.edu; 6 Neurology Department, Fortis Hospital, Bangalore 560010, India; pudukode.krishnan@fortisheakthcare.com; 7 Stroke Monitoring and Diagnostic Division, AtheroPoint™, Roseville, CA 95661, USA

**Keywords:** PD, AI, bias, mean score, cutoff, recommendations

## Abstract

Background and Motivation: Diagnosis of Parkinson’s disease (PD) is often based on medical attention and clinical signs. It is subjective and does not have a good prognosis. Artificial Intelligence (AI) has played a promising role in the diagnosis of PD. However, it introduces bias due to lack of sample size, poor validation, clinical evaluation, and lack of big data configuration. The purpose of this study is to compute the risk of bias (RoB) automatically. Method: The PRISMA search strategy was adopted to select the best 39 AI studies out of 85 PD studies closely associated with early diagnosis PD. The studies were used to compute 30 AI attributes (based on 6 AI clusters), using **AP(ai)Bias 1.0** (AtheroPoint^TM^, Roseville, CA, USA), and the mean aggregate score was computed. The studies were ranked and two cutoffs (Moderate-Low (ML) and High-Moderate (MH)) were determined to segregate the studies into three bins: low-, moderate-, and high-bias. Result: The ML and HM cutoffs were 3.50 and 2.33, respectively, which constituted 7, 13, and 6 for low-, moderate-, and high-bias studies. The best and worst architectures were “deep learning with sketches as outcomes” and “machine learning with Electroencephalography,” respectively. We recommend (i) the usage of power analysis in big data framework, (ii) that it must undergo scientific validation using unseen AI models, and (iii) that it should be taken towards clinical evaluation for reliability and stability tests. Conclusion: The AI is a vital component for the diagnosis of early PD and the recommendations must be followed to lower the RoB.

## 1. Introduction

Parkinson’s disease (PD) is a neurodegenerative disorder; James Parkinson first portrayed it in 1817 [1,2]. Globally, over 2% of the population is more than 65 years of age, and around 5–20 people per 100,000 each year are affected by this illness, demonstrating its predominance and frequency rate with maturity [3,4,5]. The registered PD cases reported in the UK were more than 1.45 million [6]. In India, approximately one million cases have had similar experiences for symptoms of PD [7]. Besides these challenges, the pharmaceutical industry has been slow in producing PD drugs. The last invention in this area was in 1967 [8].

PD illness is described by the disturbing dopaminergic cycle of the nerve cells of substantianigra [9,10,11]. A piece of the mind can create neurotransmitters such as “dopamine,” which fills in as a synapse for controlling developments in various body segments. The degenerative interaction begins from the foundation of the mind that prompts the annihilation of olfactory bulbs [12]. It is trailed by the lower cerebrum stem, affecting the susbstantianigra and mid-cerebrum [13]. Ultimately, it obliterates the limbic framework and front-facing neocortex, worsening physical and mental side effects.

**Figure 1:**
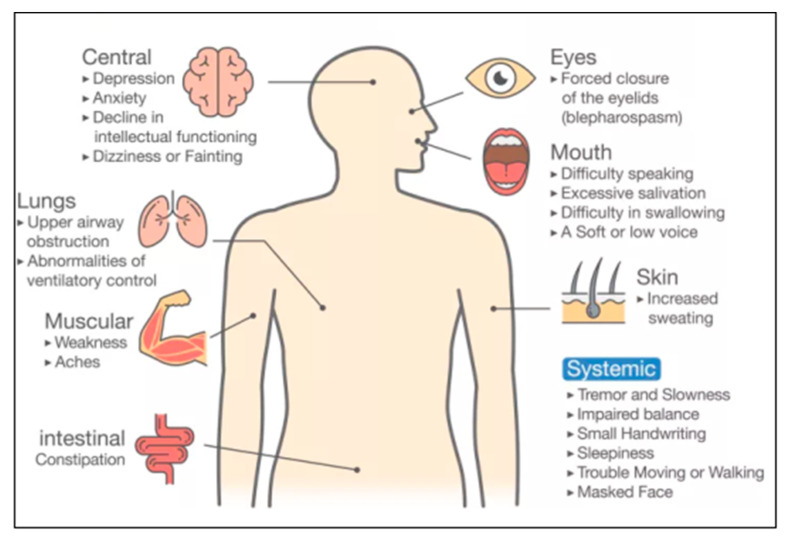
Symptoms of PD disease [37].

The symptoms related to PD can be categorized in two ways (i) verifying the patient’s PD biomarkers, and (ii) by physically observing the differential response from the patient’s body parts [14,15]. Examples of PD indications are the forced closure of eyelids during eye tests [16], lack of breathing during lung tests [17], muscle stiffness during muscle tests [2], and movement of patients while walking [10]. Figure 1 shows various PD symptoms, namely, constipation problems, feelings of anxiety, depression, and abnormalities in breathing [18]. Other symptoms include difficulty in speaking [5], voice tone changes [17], and difficulty in swallowing food [19].

Artificial Intelligence (AI) has recently dominated healthcare, particularly in medical imaging [20,21,22]. Machine learning (ML) has further enhanced the ability to accurately and swiftly make the decisions in the diagnosis of several diseases such as diabetes [23,24], stroke [25,26,27], coronary artery disease prediction [28], and cancer detection in the thyroid [29,30] liver [31], prostate [32,33], and ovaries [34,35]. Recently, there have been attempts to diagnose PD early using AI, especially using ML and DL algorithms [9,11,18,36,37]. The ML/DL algorithms are sensitive to the sample size during the training model generation, and further, due to lack of (i) scientific validation, (ii) clinical evaluation of these AI strategies, and (iii) big data configuration [36], leads to bias in the AI. Thus, when PD symptoms (or risk factors) are considered as input to the AI model, one must ensure that the AI system is reliable, accurate, and has minimal AI bias. Therefore, the primary objective is to automatically identify the AI studies that have bias. In the secondary objective, the goal is to automatically detect the studies that lie in the three categories of bias, such as low, moderate, and high bias. Further, there is a need to understand the AI architectures used in these studies and link them with the AI attributes for different categories of AI bias. Lastly, we need to identify the RoB in these AI studies and suggest possible reduction recommendations. Further, we note that the scope does not involve developing the correlation between PD and other medical conditions.

Our strategy is to score the 39 AI studies using 30 AI attributes per study with the help of an AI expert that has more than 15 years of AI experience, and then compute the mean aggregate score. Moderate-Low (ML) cutoff was determined using the intersection of the frequency plot of mean score vs. the cumulative frequency plot, where Moderate-Low (ML) cutoff was determined. Further, the second High-Moderate (HM) cutoff was computed based on the transition of slopes. The studies in low-, moderate-, and high-bias were then analyzed for recommendations to reduce the RoB.

The layout of this review is as follows. Section 2 presents the PRISMA model for selecting studies along with the statistical distributions of the parameters. Section 3 presents the AI architecture for PD diagnosis, while Section 4 presents the strategy for the computation of bias and the ranking of the studies used for bias analysis and its analysis. Finally, the critical discussions are presented in Section 5, leading to conclusions in Section 6.

## 2. Search Strategy and Statistical Distribution

### 2.1. PRISMA Model

An end-to-end writing search was performed utilizing PubMed, IEEE Xplore, Science Direct, and Google scholar. The significant watchwords utilized for choosing these studies were PD disease, neurodegenerative disease and symptoms, AI, machine learning, and differential finding of the neurodegenerative disease. The research articles selected for the studies consist of various parameters like detections of the PD by using machine learning, deep learning, hybrid learning, and AI. These research articles have also shown the classification of the normal vs. PD-affected people, the demographic analysis of the PD-affected patients, and the classification of the PD by considering the input parameter alternative assessment as one method to detect PD [9]. Studies unrelated to the symptomatic observation of PD are eliminated in published papers for many reasons [11,12,38]. Therefore, studies that are not related to the symptomatic observation of PD are excluded [39].

**Figure 2:**
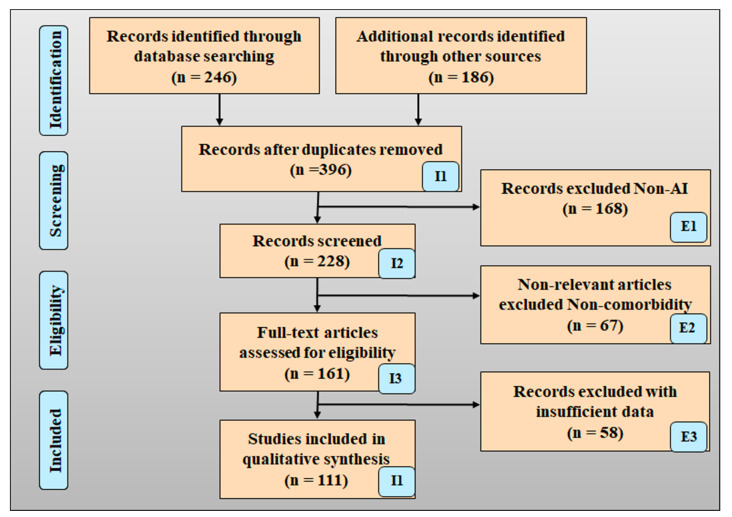
PRISMA model for early detection of the PD by using AI.

Figure 2 shows the PRISMA model for the selection strategy of the research articles. The identification phase shows that nearly (246) articles were searched from the identified sources, and 186 studies were searched from the other sources. A total of 396 study articles were removed as they cross the study objective and have duplications. Considering the feasibility of the objective of a selection strategy (396 studies), the articles were screened. The non-AI-based total (168 studies) articles were removed. Many discuss irrelevant information other than the objective of the search strategy. Most of the articles do not fulfill domain criteria like lack of data, lack of information, and poor presentation of the articles. Hence, the total (103 studies) studies are referred to for the analysis [40].

A study that does not include input parameter analysis, performance optimization, attributes analysis, and benchmarking was also not evaluated. Alzheimer’s disease, Huntington’s disease, Motor neuron disease, and Adrenoleukodystrophy (ALD) disease are not categorized as PD. Studies not performed in humans (rat, monkey, etc.) were excluded, as well as studies that do not have a huge sufficient dataset for analysis. The primary objective was to automatically identify the AI studies that have bias. In the secondary objective, the goal was to automatically detect the studies that lie in the three categories of bias, such as low, moderate, and high bias. Other exclusion criteria included having no correlation between Parkinson’s disease with other neurological diseases mentioned in the manuscript, and if the article was written in a different language other than English [1,41]. The information considered for the PD studies’ data extraction was (i) author name, (ii) year of publication, (iii) objective of the studies, (iv) demographic discussion, (v) data types, (vi) data source, (vii) diagnosis method, (viii) bias studies, and (ix) attribute studies. The selected studies were evaluated with the novel and unique implementation of the AI, hybrid AI, twin diagnosis approach, telemedicine approach, and biomarker-based approach for diagnosing PD. Every study was evaluated with feasibility analysis and cross verified with scientific validation [42].

**Figure 3:**
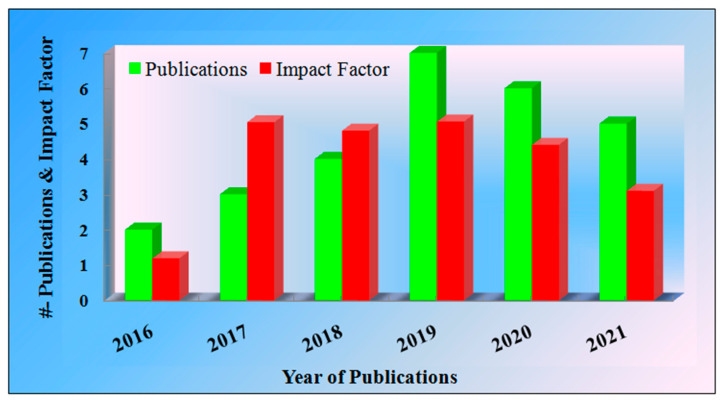
Year of article publication vs. impact factor.

Figure 3 represents the year of publications with reference to the impact factor. From the analysis point of view, we considered publications from the period 2016 to 2021. While observing Figure 3, it is clear that in 2019, the maximum publications are related to the early detection of PD and have a good impact factor. The use of datasets from open-source repositories to minimize research costs also leads to improved performance and overall applicability of the selected model. There is the risk of the bias coming out as High-Moderate (MH) if the model fails to adopt the appropriateness of the open-source data.

### 2.2. Statistical Distribution

The study objectives include the term exposure of “Parkinson’s disease.” The statistical distribution of the selected studies separates the main AI terms into ML, DL, and HDL [11,12]. The majority of the studies used ML for PD detection, and this accounts for 74%, while 9% use SDL [43,44,45] and 17% use HDL. The performance indicator of the selected algorithm plays a crucial role in bias estimation. Even though accuracy is good, there are chances of the existence of bias or inclusion bias due to the non-clinical validation of AI-based predictions [46,47,48].

#### Performance Metrics

Symptoms (or risk factors) of PD are considered as input to the AI model. It is important to ensure that the AI system is reliable, accurate, and has minimal AI distortion. The ML/DL algorithm is sensitive to sample size during training model generation and lacks scientific validation and the clinical evaluation of these AI strategies, resulting in a bias in the model.

**Figure 4:**
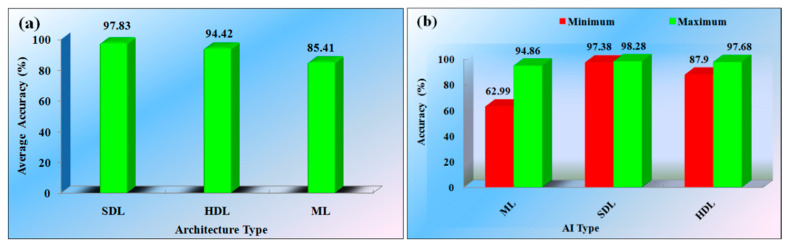
(**a**) Average accuracy of the various architectures for PD. (**b**) Minimum (red) and maximum (green) accuracy of the different PD architecture (AI: Artificial Intelligence, SDL: Solo deep learning, ML: Machine learning, HDL: Hybrid deep learning).

The SDL (2 studies) architectures were used to detect the PD, showing an average overall accuracy of 97.83%. The maximum accuracy was 98.28% and the minimum was 97.38% for the SDL architecture. The HDL (4 studies) represent the average accuracy of 94.42%, while the maximum accuracy was 87.90% and the minimum was 97.68%. The ML-based model (17 studies) showed an average accuracy of 85.41%. The maximum observed was 94.86% and the minimum was 62.99%. Figure 4a,b, respectively, indicate the average accuracy of the studies and minimum and maximum accuracy of the individual studies.

**Figure 5:**
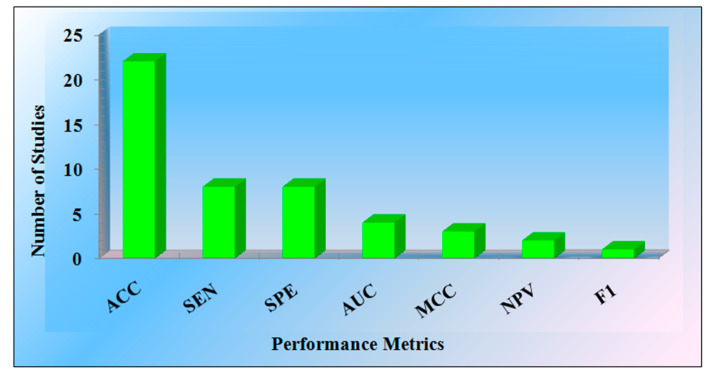
The performance metrics vs. the number of studies (ACC: Accuracy, SEN: Sensitivity, SPE: Specificity, MCC: Matthew’s correlation coefficient, NPV: Net present value, F1: Dice similarity coefficient).

It is clear from the analysis of AI-based studies that the DL models provide the highest accuracy, and then comes the HDL and ML-based studies [14,20,49,50,51]. Various models are accessed to evaluate the performance of the studies. Most studies comment on the model’s accuracy. Few of them represent the sensitivity, specificity, area-under-the-curve (AUC), net present value (NPV), and F1-score. Figure 5 represents the graph of the performance metrics versus the number of studies.

Table 1 represents the 22 studies’ comments on the accuracy parameter, with eight studies representing evaluation in terms of sensitivity and specificity, and the parameters AUC (4 studies), MCC (3 studies), NPV (2 studies), F1 (one study) were mentioned in the research articles.

**Figure 6:**
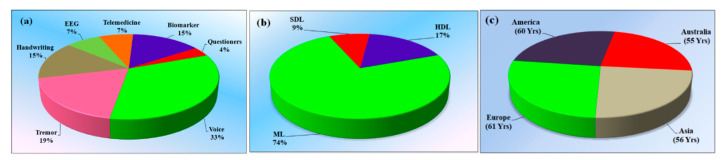
(**a**) Various kinds of input for the detection of the PD, (**b**) types of AI architectures, and (**c**) demographics of PD patients in four continents (SDL: Solo deep learning; ML: Machine learning; HDL: Hybrid deep learning).

Out of a total of 29 studies, 9 studies (33%) used voice as input parameter for the detection of the PD, 5 studies (19%) used tremor data, 4 studies (15%) used sketch as the input parameter, 2 studies (7%) used EEG, and 2 studies (7%) uses telemedicine for diagnosis. From the studies, it is fair that the input parameter is the crucial factor for diagnosing the disease [3,52,53,54]. Figure 6a indicates the various distributions of the input dataset features for the diagnosis of PD. Figure 6b refers to the statistical distribution of the selected studies, which separates the main AI terms into the machine, deep, and hybrid studies. The input element for early predication of PD is important for the reckoning of bias in the studies.

## 3. Biology of Parkinson’s Disease

**Figure 7:**
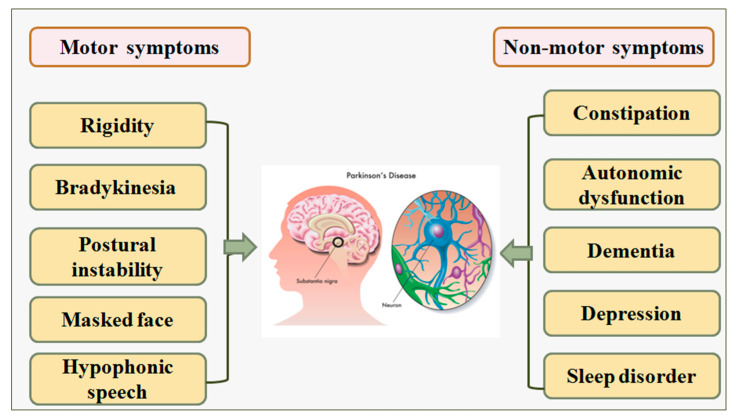
Symptomatic biology of PD.

The declension of nerve cells in the substantianigra region of the brain causes PD. This part of the brain is responsible for producing a neurotransmitter called dopamine, which is originated by nerve cells. The role of dopamine is to act as a mediator between the brain and the elements of the sensory organs that govern and regulate physical movements [20]. The abundance of dopamine in the brain is lowered when these neurons die or become injured. This indicates that the part of the brain that controls movement cannot function correctly, resulting in slow, unwanted, and irregular movements of the body parts [55]. The death of nerve cells is a gradual process. When somewhere around 80% of the nerve cells in the substantianigra are damaged, signs of PD begin to appear [56]. Figure 7 depicts the clinical biology of Parkinson’s disease [38,57]. Although additional research is desired to find the exact cause for the loss of nerve cells associated with PD, there are no proper explanations for why it happens [3,11]. The cause of the disease is currently linked with a mix of environmental factors and genetic mutations. Several hereditary variables have been demonstrated to enhance a person’s risk of getting PD, while it is unidentified how these factors make certain people more susceptible to the disease [53,55].

The abnormal genes are transferred down from parents to children, and PD can run in families. However, this is a rare kind of legacy for the condition. According to some experts, environmental variables may also enhance a person’s risk of PD [57]. The use of pesticides and herbicides in agriculture and industrial pollution and traffic have been suggested as impactable causes to trigger PD. The data relating external factors with PD, on the other hand, are ambiguous [41,58].

The motor symptoms (risk factors) of PD can be used for classification (PD vs. Non-PD) using the AI-based model. The dataset was generated while evaluating the patients and can be easily put in a matrix form to develop the training model. The huge PD symptomatic data are generated including motor and non-motor PD risk factors. The symptomatic data cannot be statistically resolved, but an ML/DL/TL/HDL can be used to better understand both data classifications, leading to better PD detection [59]. While analyzing the symptomatic biology of PD, in-feature AI is the best option to quickly predict PD.

## 4. Artificial Intelligence Architectures

The Artificial Intelligence (AI)-based detection of the PD can be achieved by using symptoms (or risk factors) as an input parameter for the algorithm. The majority of the studies explain voice as a risk factor for the diagnosis of PD [52]. Tremor data are also an important (risk factor) in detecting PD [6]. The hybrid model includes two risk factors, which were also explained in a few articles [14,60].

### 4.1. A Note on Assumptions for Adaptation of the ML Algorithms

Different input parameters brought different assumptions. When the input is a tremor, if the shaking is prevalent in one body part (say the uncontrolled movement of hand), HDL such as ANN was preferred. Since NN could handle the augment, scale, and normalization, it preferred HDL. In the case when the input data was a voice, ML was preferred. In the case of voice datasets, the main assumption for the application of ML was to help diagnose the early and subtle signs of PD. In other cases, since the gold standard was available, the assumption was that the training models can be very powerful for the early diagnosis of PD. A certain set of ML algorithms such as principal component analysis was adopted due to a reduction in the dimension of the input datasets.

The features of the voice database can be better analyzed using decision tree or k-mean clustering methods, and such classifiers can be better suited for voice data classification for control vs. PD. Since the voice data were violating the data in components, it was assumed that by breaking the voice data into components and then feeding it into ML algorithms, such as hidden Markov models, then the learning of the voice data can be the superior method, followed by the detection process. A deep convolutional neural network classifier with transfer learning and data augmentation techniques can be used to identify the risk of the PD. The usage of handwriting data for the prediction of the PD faces a severe classification challenge at the preliminary stages due to the small size of data. The use of ImageNet and MNIST datasets were used as input sources independently to achieve good accuracy. For accurate identification of PD, other parallel PD symptoms data such as voice, freezing, and gait can be used

### 4.2. Architecture Based on Voice and Sketch Input

**Figure 8:**
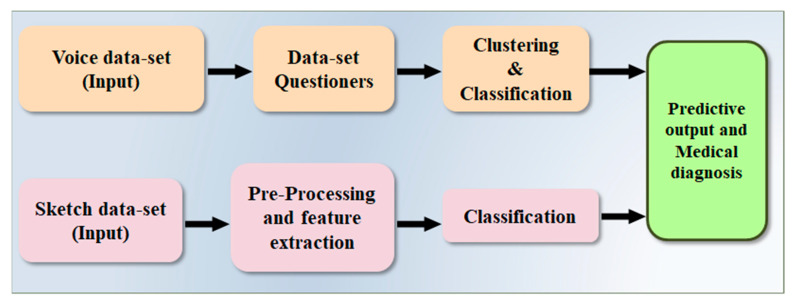
Proposed methodology for early detection of PD by Anitha et al. [37].

Anitha et al. [38] proposed a methodology (Figure 8) to predict PD using a clustering and classification algorithm. On the voice dataset, k-means, clustering and decision tree-based ML algorithms are evaluated using R-studio. Python is used to analyze the patient’s spiral artwork. Principal component analysis (PCA) is used to extract features from these illustrations. X, Y, Z, Tension, Grip Angle, Timestamp, and Test ID variables are derived from the spiral drawings. For comparison, two factors are used for the UCI dataset and drawing the data. In the study, the accuracy was demonstrated to be 76% and 91%, respectively. In comparison to other literature, the accuracy is low. It is feasible to improve the model’s accuracy by combining DL with an existing algorithm [38].

### 4.3. Architecture Based on Tremor

**Figure 9:**
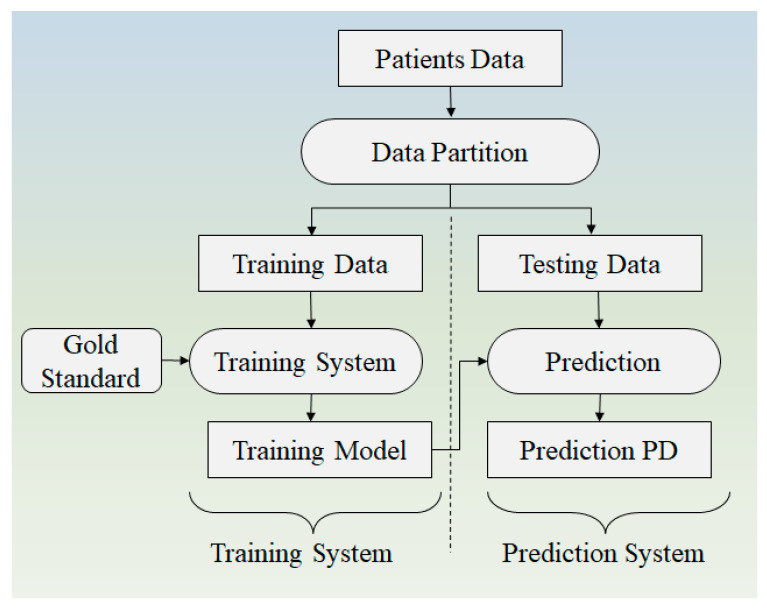
Proposed architecture for early detection of PD [6].

Bala et al. [6] have proposed architecture for early detection of PD by using ML-based classification methodology (Figure 9). Two types of data elements used for analysis were the tremor dataset and speech dataset. Data of 77 (PD) patients were used for experimentation purposes. By using the computer algorithm Multi-Dimensional Voice Program (MDVP), 33 acoustic parameters of a voice sample were calculated. The program that can calculate various algorithms such as K-mean, Random Forest, SVM, NB, and KNN is applied to the dataset. In both cases, accuracy was calculated for speech signal using NB (88.05%) and for tremor by using KNN (85.67%). The detailed design does not discuss any standard database used [6].

### 4.4. Architecture Based Speech Input with Information Gain Parameter

**Figure 10:**
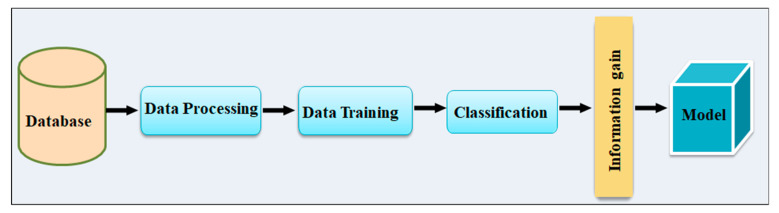
Proposed Architecture for early detection of PD by Cleick et al. [61].

To predict PD, Cleick et al. [61] presented a variety of classification methods, including Regression analysis, Support Vector Machine, Extra Trees, Gradient Boosting, and Random Forest (Figure 10). In the classification stage, a total of 1208 voice data sizes were employed, with 26 features gathered from PD patients and non-patients. Classification results obtained using enlarged features beat classification obtained results using the data’s unique features. Random forest was used to get an IG accuracy of 72.69 percent [57].

## 5. Ranking of Selected Studies

Since some studies offer better AI model designs than others for early PD detection, it is important to understand which studies are more suitable for early PD detection. For this objective, one must rank these studies and evaluate the bias in their AI models. These studies can then be partitioned into certain bias bins, which can have their own AI characteristics. Note that the AI model performance is governed by the AI architecture and its components (so-called AI attributes). Thus, a study must have an evaluation criterion by which one can grade these AI attributes, which can then be used for evaluation or ranking.

The various architectures in the studies explain the role of AI in the detection of PD [55,59]. If the components of the AI architecture used for early detection of the PD have low performance, then the AI models under-performs, leading to lower grading of that study [57]. Attribute studies, combinations of the input parameter, and benchmarks associated with the clusters of the studies are essential factors that decide the ranking of the studies [55,56,58]. The detailed subsection explains the various parameters related to the raking of the studies.

### 5.1. Grading, Scoring, and Ranking of the Studies

Every study graded correlated with the attributes; a total of 30 attributes were considered for evaluation purposes and clustered into six sections. The cluster (C1) is related to publication and citation, (C2) is about the objective of the studies, (C3) explains the types of AI architecture used in the model, (C4) demonstrates optimization of the AI algorithms, (C4) analyzes the performance and evaluation of various AI models, (C6) is about clinical evaluation, scientific validation, and benchmarking. Every attribute in the respective cluster was evaluated for the evaluation purpose grading score method, as explained in Table A1 (Appendix A).

After interpreting the results of every cluster of the associated studies (26 studies) mean value, the absolute score cumulative score was computed. According to the mean value, absolute score, and cumulative score of the concerned studies, the ranking of the studies was finalized. The ranking studies are mentioned in Table 2 [61,62]. The green, yellow, and red flags indicate the impact of low-bias, moderate-bias, and high-bias on individual cluster cells.

### 5.2. Bias Cutoff Computation

About 26 studies were selected for the bias analysis that was closely associated with early detection of PD. Using **AP(ai)Bias 1.0** (AtheroPoint^TM^, Roseville, CA, USA), bias analysis was carried out. Studies were ranked into three AI bias categories (low moderate (ML) and high moderate (MH)) by computing the mean score and cumulative score for each study, taken for the AI attributes. The comparative analysis with various AI algorithms was carried out to determine the bias cutoff and to understand the architecture of these studies [59,63,64].

It is seen that many of the AI models show high accuracy, but the data size used for the testing and training of the algorithm is small, and the model fails to explain scientific validation. Hence, it results in High-Moderate (HM) in the studies [1,5,9,37,62,65]. The cumulative cutoff for the studies was determined by using various factors such as (i) associated studies of the PD, (ii) impact factor, (iii) the selected data, (iv) performance indicators, (v) clinical trials, etc. After analyzing the selected studies (26 studies), the cutoff was finalized for the high-bias <0.064 (8 studies), moderate-bias <0.078 (8 studies), and low-bias >0.078 (7 studies).

**Figure 11:**
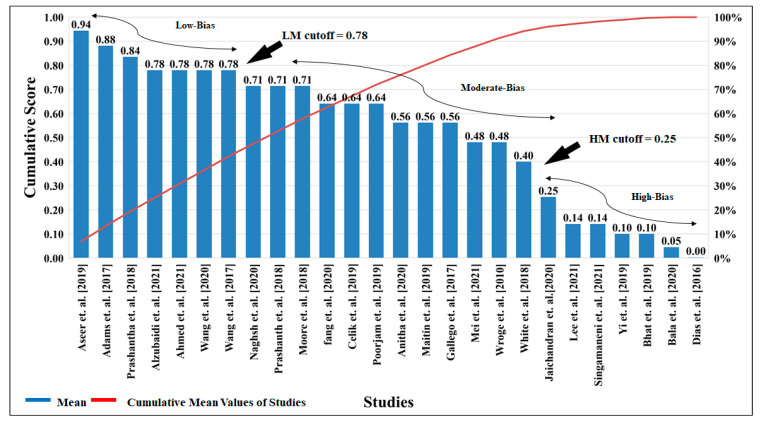
The cumulative cutoff score for evaluation for the selected studies. (LM: Low-Moderate, MH: High-Moderate).

The Low-Moderate (LM) studies [1,5,9,37,62,65] observations are the articles containing information such as (i) high data count of the PD vs. normal; (ii) performance measures; (iii) comparative analysis with various ML, DL, and HDL algorithms; (iv) explanations of the benchmarking studies. The Moderate-bias studies [1,5,9,37,62,65,66] observations were (i) sufficient data, (ii) average impact factor, and (iii) comparison of the input parameters. The High-Moderate (HM) studies [3,6,54,60,67,68] observations associated with the articles were (i) a smaller number of data, (ii) insufficient dissuasion on the selected model, (iii) improper explanation of the algorithm, (iv) insufficient performance analysis, (v) lack of demographic discussion, and (vi) insufficient discussion on clinical evaluation. Based on the attribute analysis, every cluster was marked. The benchmarking and attribute analysis were not done. The algorithm with classifier optimization was not explained [15]. There are several explanations as to why and how the articles were frittered away for the research [63]. Figure 11 shows the cumulative cutoff score for the evaluation of the selected studies.

While noting the ranking studies, it is clear that selecting the architecture model for the proximate input is essential. It is linked with the performance of the model and RoB [37,69,70]. In the case that more than one input was taken for the diagnosis of the PD, the architecture paradigm and the performance of the model would change [49,68]. Hence, it is essential to discuss the linking of the architecture concerning input parameters for diagnosing PD [18,71,72]. Table A3 (Appendix C) discusses twelve studies linked with AI models’ performance parameters and compared them with input risk factors.

### 5.3. Linking of Bias with AI Architectures

**Figure 12:**
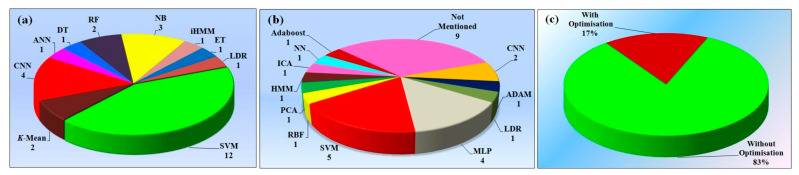
(**a**) Various types of architectures were mentioned in the studies; (**b**) classifier used; (**c**) optimization studies.

The various databases contain the resultant features of the voice, sketch, tremor, face, EEG, and a biomarker of the PD patients concerning the normal [73]. UCI, PubMed, IEEE, and MJFox are the few names of the database providers. Some of the articles also include local datasets for the analysis of PD [60,74]. Figure 12a represents the various algorithms used for the detection of the PD studies. The SVM algorithm, along with Decision Tree, Naive Bias, and Random Forest, was used. Few articles compare various algorithms with each other and compare their performance evolutions [12,70,75].

Table A2 (Appendix B) explains the various statistical significance of the input features selection for the diagnosis of the PD and the performance parameter of various AI architectures [19,76]. The architecture uses a model with a classifier. Optimization was discussed in the third cluster. The fourth cluster related to evaluating the performance includes parameters such as accuracy, AUC, MCC, and F1. The evaluation and benchmarking sections discussed seen unseen data, as well as conformability of the data. Table A3 (Appendix C) represents the attribute analysis [67]. The basic model of AI consists of (a) PD vs. normal training and (b) risk label forecasting (risk possibilities) on test scenarios. As a result, these learning methods were categorized according to the type of results (scoring element) of the models, the category of classifiers, the clusters of predictor variables (risk factors), the predictive unbiased for the short or long term, the type of cross-validation procedure, scientific validation, and the outcome diagnosis. These aspects are crucial in determining performance as well as hazards that lead to bias.

### 5.4. Bias Distribution in AI Attributes

The tri-color scheme was implemented to represent the scientific analysis for low, moderate, and high-bias in the various attributes of the clusters. The Low-Moderate (LM) observations were done for articles containing information such as (i) high data count of the PD vs. normal; (ii) performance measures; (iii) comparative analysis with various ML, DL, and HDL algorithms; (iv) explanations of the benchmarking studies; (v) Implantation of the PRISMA model search strategy. The High-Moderate (HM) studies [3,6,54,60,67,68] observations associated with the articles were (i) less numbers of data, (ii) insufficient dissuasion on the selected model, (iii) improper explanation of the algorithm, (iv) insufficient performance analysis, (v) lack of demographic discussion, (vi) no comments on clinical evaluation, and (vii) unmentioned benchmarking of the attribute. It is observed in the bias distribution studies plot that most of the articles do not discuss the clinical evaluation and benchmarking, which lead to an increase in the high bias of the selected studies [3,6,54,60,67,68].

**Figure 13:**
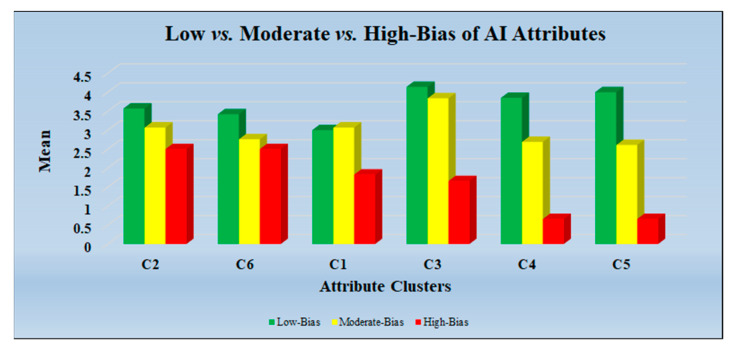
Low vs. Moderate vs. High-Bias of AI Attributes (C1: Citation, C2: Objective and Design Methodology, C3: AI Architecture, C4: Optimization of AI Model, C5: Performance Evaluation of AI Models, C6: Clinical Evaluation and Benchmarking).

The insufficient optimization of the AI architectures with many inputs also leads to high bias. The good accuracy of the AI model but with failed test clinical validation results also leads to high bias. The comparative analysis with various AI algorithms was carried out to determine the bias cutoff and to understand the architecture of these studies. The cluster-wise bias distribution plot is shown in Figure 13.

### 5.5. Recommendations for Bias Reduction

The recommendation is an integral part of the study evaluation. We summarize the key recommendations, which can potentially improve the bias in AI for early PD detection, namely (i) Validation: the AI-based PD detection should be scientifically validated and clinically evaluated [39,52,77]; (ii) Fusion of covariates: is recommend that the AI model uses combinations of risk factors as an input parameter for the detection of PD [40] to ensure non-linearity is detected; (iii) Continental databases for AI generalization: use of the “continental multiethnic categorized dataset” and usage of power analysis (in big data framework), which will lead to improving true accuracy of early PD predication [72,78,79,80]; (iv) Non-motorized symptoms: “non-motor validated data” for PD (risk factors) data (Figure 7) are important risk factors for the AI models and must be included [58,81,82,83]; (v) Comorbidities: the PD risk factors due to “comorbidities” like COVID-19 [59,84,85,86,87], diabetes [23,24], and liver [88,89,90], thyroid [91,92], coronary [32,93,94], prostate [95], ovarian [96], and skin cancer [97,98] must also be considered.

## 6. Discussion

### 6.1. Principal Findings

PD is a non-curable disease, but at an early stage with a correct and precise diagnosis, we can control the progression of the disease. AI is a good option to detect an early-stage PD compared to the conventional PD detection approaches. However, there is a risk of bias in AI models due to lack of AI design attributes, which also includes gold standards (risk factors) of PD. This proposed review is the first to discuss AI bias analysis in the early detection of PD. As a result of this study, the outcomes are (i) Usage of computing 30 AI attributes (based on 6 AI clusters) scored by an AI expert, and computation of mean aggregate score; (ii) Computation of two cutoffs (Moderate-Low (ML) and High-Moderate (MH)) and determination of three bins: low-, moderate-, and high-bias. Additionally, (iii) it is seen that many of the AI models show high accuracy but the sample size used for the testing and training of the algorithm is relatively small. Further, the model fails to explain scientific validation; hence, it results in High-Moderate (HM) bias in the studies. (iv) For an AI system to be reliable, accurate, and to have a minimal AI distortion, the bias must be minimal. (v) AI architecture such as deep layered neural network models and such as the ANN model were neglected in clinical design and decisions (e.g., voice, tremor, sketch) and indicate Moderate-Low (ML) bias in the ranking [13,62,99].

### 6.2. Benchmarking

Table 3 shows the benchmarking analysis of the eight selected AI studies. We have also mentioned various important aspects of the review related to early PD detection by using AI [100,101]. The demographic analysis of the PD is mentioned in column (B3). While analyzing demographics, we can find important factors such as the continent/country that is leading and lagging in major/minor cases of PD patients [43,102]. The (B4) column benchmarking table represents the objective of the studies. Most of the studies represent the comparative analysis of a normal person to a person diagnosed with PD [54]. Column (B4) is related to the inclusion and exclusion criteria of the studies. As per the disease symptoms point of view, most of the symptoms under the tree of neurodegenerative diseases such as Alzheimer’s, Huntington’s, Adrenoleukodystrophy (ALD), and PD are similar [37,43,58]. Few symptoms of the disease among them are different. When selecting the articles for the proposed study, we tried focusing on the symptoms related to PD [103,104]. As mentioned in column (B5), the data extraction criteria from the various sources are important to focus on the area of interest in the study [51,104]. The various AI models used in the studies are mentioned in column (B6), and it has been seen that in most of the article, ML algorithms were used to detect PD. The performance of various AI models is shown in Table A3 (Appendix C) [63]. The studies using the PRISMA model strategy for selecting the article were verified and are shown in column (B7) [4,32]. Column (B9) represents the risk factor as an input parameter analysis for the early detection of the PD. The early symptoms of PD are compulsiveness in movement, voice changes, and movement problems [4,16,17]. It is easy to predict the disease by observing the change in motion of the body parts such as freezing of the shoulder [6,14]. The column (B10, B11, B12, B13, and B14) represents benchmarking observations, cross-validation, bias studies, and scientific validation, respectively, but most of the selected studies failed to explain those terminologies. The last row depicts “Proposed,” which is about the current study. Note that we indicated “√” in places of solitary benefaction in the review.

### 6.3. A Short Note on Bias in ML

PD is a non-curable disease, even though the treatment cost of PD is very high. To avoid death and economic loss due to the late diagnosis of PD, early diagnosis of PD is very important. AI is a good option to detect the early stage of PD compared to the conventional PD detection approach, but compared to the conventional PD detection approach, there is a risk of implementing an AI model. An AI model is evaluated based on accuracy only, but the model fails to explain scientific validation and clinical validation. Further, there is a lack of evidence on the generalization of AI models; hence, it results in High-Moderate (HM) bias in the AI model. Many of the AI models show high accuracy, but the data size used for the testing and training of the algorithm is small; thus, it results in High-Moderate (HM) bias in the AI model. The AI-based detection of PD can be achieved by using symptoms (or risk factors) as an input parameter for the algorithm. The majority of the studies explain voice, tremor, gait, and sketches as risk factors for the diagnosis of PD [14,52,60]. It is seen that the AI model uses combinations of risk factors as the input parameter for the detection of PD, having a Low-Moderate bias.

The studies were used to compute 30 AI attributes (based on 6 AI clusters). The PD risk is intensifying due to existing comorbidities with PD; hence, it results in High-Moderate (HM) bias in the AI model. By adding more attributes such as comorbidities with PD, gender studies of PD patients, and clinical validation of AI-assisted PD detection, the grading score of the studies will be improved. Therefore, there is scope to minimize the High-Moderate bias in the AI model [83,102].

### 6.4. A Short Note PD Database and Gender Studies

Figure 12c shows the demographic distribution of the various continents, American (60 years), Europe (61 years), Australian (55 years), and Asian (56 years), and the average age of the PD patients in these respective continents [102,105,106].

Furthermore, their risk of granularities of a database to predict the PD results High-Moderate (HM) bias in the AI model. As lifestyle, environmental conditions, and human factors vary with the continents, attributes of the dataset will also vary. Thus, the unavailability of the continental categorized dataset of the PD AI model leads to High-Moderate (HM) risk. The average age of the PD patient is 57.77 years, and most of the database contains the age group of the patients between 50 to 60 [8,102]. Hence, the majority of risk factors are probably affecting PD patients in the age group of 50 to 60 years [59,75,107]. The PD risk is intensifying due to existing comorbidities. If we eliminate the associated comorbidities with PD to train the model, it results in High-Moderate (HM) bias in the AI model. There is no study of Age/Gender in certain ethnicities, and without this, the bias will erupt. Such a system that has not included the diversity in age will fail in the prediction models if the training is not also correct, so there is the risk of generating high bias in the model.

### 6.5. Role of Human-Computer Interface in Early Detection of the PD

Human-computer interaction (HCI) studies the interaction among humans and computers, providing indicators that may be used to assess a user’s physiological, behavioral, and psychological states, for example. Computers, cellphones, tablets, gaming platforms, and wearable technologies all fall under the heading of human-computer interaction (HCI). By using HCI, it is easy to predict early PD motor symptoms, for example, by monitoring the keyboard or touch screen of smartphone operating response from the user. There seem to be a variety of features present during typing on a keypad, according to current studies on PD diagnosis through different motor symptoms, including reaction speed to messages, uneven movement of the figures, typing pattern, degradation of repetitive movements, stiffness in figures, indications of sidedness, deterioration in repetitive motion and typing of sequences of letters, changes of motion and signs of hand and finger muscle spasms, and Jerkiness of movement. Therefore, the HCI parameters can be considered for the early detection of PD [1].

### 6.6. Strengths, Weakness, and Extensions of Our Study

The main strength of the study is the ability to automatically compute the RoB given the scored AI attributes by an expert in the AI field. These attributes were an amalgamation of demographics, AI architecture, performance evaluation, scientific validation, clinical evaluation, and big data analysis, framed into six clusters [108,109]. The second component was to compute the aggregate score for each of the AI studies, followed by an estimation of two cutoffs (Moderate-Low (ML) and High-Moderate (MH)) to classify the studies into three bins: low-, moderate-, and high-bias. The study further provides new insight into the building blocks of AI-based early PD detection such as architectural differences, input risk factors, and limited databases, which are the key elements responsible for RoB in the AI model. Further, the study presented a set of key recommendations for improving the RoB. The studies lacked discussions on database size, comorbidities with PD, gender information in PD, continental databases, and clinical validations of AI-assisted PD detection. By adding relevant, meaningful, and quality attributes to benchmarking, the RoB of the AI model may also be improved [7,20,86,110]. Some studies may help to observe the PD study of problem-solving and executive function.

Due to a lack of research funding and the non-involvement of the leading worldwide groups in the field of AI, the benchmarking section was compromised in quality. Even though it was a pilot study, due to a lack of AI participation in the PD field, the RoB has the potential for exhaustive analysis. Further, due to the COVID-19 pandemic, the PD research funds are limited and, therefore, PD research has been less attractive [86,111].

We expect to see more systematic reviews using DL and HDL models. Further, other neurological diseases such as Alzheimer’s and Adrenoleukodystrophy (A.L.D.) [112,113], when aligned to PD, can be explored for more robust scoring, ranking, and classification using advanced neural imaging tools [69,114,115]. Currently, the world is facing a COVID-19 pandemic, where 26 million people are affected and 5.2 million have died due to the coronavirus. COVID-19 has strongly affected neurological diseases due to its brain pathway [11,116]. Further, several comorbidities like diabetes, renal disease, and coronary artery disease have intensified in COVID-19 patients, causing pulmonary embolism [59,111]. Several AI tools have been researched and recommended for COVID-19 applications [86,117]. Just like one can characterize the lung or pulmonary COVID-19 data [110,118], there can be PD neurological imaging data on COVID-19 patients that can be analyzed. Recently, bias estimation on COVID-19 patients was designed and developed [59]. In the future, we anticipate more systematic reviews on PD-based RoB with comorbidities focusing on the COVID-19 virus [59,84,85,86,87].

## 7. Conclusions

To our knowledge, this is the unique review that contains RoB elements selected from all 26 research articles that used machine-learning, solo deep-learning, and hybrid-learning algorithms to diagnose PD. We shared our findings, which included studies in a high-level summary, such as (i) the AI is an essential component for the diagnosis of the early PD detection and the recommendations must be followed to lower the RoB; (ii) the studies were ranked and two cutoffs (Moderate-Low (ML) and High-Moderate (MH)) were determined to segregate the studies into three bins: low-, moderate-, and high-bias); (iii) clinical, behavioral, and biomarker data categories were useful while verifying symptoms of the PD; (iv) possible patients biomarkers and physical indicators that are very important for making a more accurate diagnosis for helping healthcare decision-making. We recommend (i) the usage of power analysis in big data framework, (ii) that it must undergo scientific validation using unseen AI models, and (iii) further adaptation in clinical evaluation for reliability and stability tests.

The accomplishment of AI-assisted PD diagnosis holds great promise for a more systematic clinical decision-making system, and the use of innovative biomarkers would lower the bias and make it easier to understand drugs. Diagnosis of PD at an early onset will be feasible and faster with the help of AI techniques. Approaches to AI may give clinicians more valuable information for screening, detection, and diagnosis techniques towards the early detection of PD disease.

## Figures and Tables

**Table 1:** Performance metrics of selected studies.

Performance Metrics	ACC	SEN	SPE	AUC	MCC	NPV	F1
**Number of Studies**	22	8	8	4	3	2	1

ACC: Accuracy, SEN: Sensitivity, SPE: Specificity, MCC: Matthew’s correlation coefficient, NPV: Negative predictor value, F1: Dice similarity coefficient.

**Table 2:** Ranking of the selected studies.

*Low-Bias*		*Moderate-Bias*						*High-Bias*			
SN	Author	C1	C2	C3	C4	C5	C6	Mean	Absolute Score	CDF	Rank
1	Aseer et al. [1] (2019)	3	4	5	4	5	4	4.17	25	0.94	1
2	Adams et al. [2] (2017)	4	4	4	4	4	3	3.83	23	0.88	2
3	Prashantha et al. [3] (2018)	3	4	4	4	4	3	3.67	22	0.84	3
4	Alzubaidi et al. [4] (2021)	3	4	4	3	3	4	3.50	21	0.78	4
5	Ahmed et al. [5] (2021)	2	3	4	4	4	4	3.50	21	0.78	5
6	Wang et al. [6] (2020)	3	3	4	4	4	3	3.50	21	0.78	6
7	Wang et al. [7] (2017)	3	3	4	4	4	3	3.50	21	0.78	7
8	Naghsh et al. [8] (2020)	3	3	4	4	3	3	3.33	20	0.71	8
9	Prashanth et al. [9] (2018)	3	3	4	3	4	3	3.33	20	0.71	9
10	Moore et al. [10] (2018)	3	3	4	3	4	3	3.33	20	0.71	10
11	Fang et al. [11] (2020)	2	2	4	4	4	3	3.17	19	0.64	11
12	Celik et al. [12] (2019)	4	4	4	3	2	2	3.17	19	0.64	12
13	Poorjam et al. [13] (2019)	4	3	4	2	3	3	3.17	19	0.64	13
14	Anitha et al. [14] (2020)	3	3	4	2	3	3	3.00	18	0.56	14
15	Maitín et al. [15] (2019)	3	4	4	3	2	2	3.00	18	0.56	15
16	Gallego et al. [16] (2017)	2	3	4	4	2	3	3.00	18	0.56	16
17	Mei et al. [17] (2021)	2	3	3	3	3	3	2.83	17	0.48	17
18	Wroge et al. [18] (2010)	3	3	4	2	2	3	2.83	17	0.48	18
19	White et al. [19] (2018)	4	3	3	2	1	3	2.67	16	0.40	19
20	Jaichandran et al. [20] (2020)	4	3	4	0	1	2	2.33	14	0.25	20
21	Lee et al. [21] (2021)	4	4	0	0	0	4	2.00	12	0.14	21
22	Singamaneni et al. [22] (2021)	1	3	3	2	1	2	2.00	12	0.14	22
23	Hu et al. [23] (2019)	1	2	2	1	2	3	1.83	11	0.10	23
24	Bhat et al. [22] (2019)	3	3	3	0	0	2	1.83	11	0.10	24
25	Bala et al. [24] (2020)	1	2	2	1	1	2	1.50	9	0.05	25
26	Dias et al. [10] (2016)	1	1	0	0	0	2	0.67	4	0.00	26

C1: Citation, C2: Objective and Design Methodology, C3: AI Architecture, C4: Optimization of AI Model, C5: Performance Evaluation of AI Models, C6: Clinical Evaluation and Benchmarking, CDF: Cumulative score.

**Table 3:** Benchmarking scheme for selected and proposed studies.

B0	B1	B2	B3	B4	B5	B6	B7	B8	B9	CB0	B11	B12	B13	B14
SN	Citation (Year)	OB	DD	IEC	DE	ME	PM	AA	IPA	BA	CV	BS	SV	RS
1	Ahlrichs et al. [25] (2013)	Ns vs. PD	×	×	×	×	×	×	√	×	×	×	√	**72**
2	Bind et al. [26] (2015)	Ns vs. PD	×	×	×	×	×	×	×	×	√	×	×	**52**
3	Maitín et al. [15] (2020)	Ns vs. PD	×	√	×	√	√	√	×	√	×	×	√	**37**
4	Anila et al. [27] (2020)	Ns vs. PD	×	×	√	×	×	×	√	×	√	×	×	**37**
5	Watts et al. [28] (2020)	Ns vs. PD	×	×	×	×	×	×	√	×	×	×	×	**109**
6	Garg et al. [29] (2021)	Ns vs. PD	×	×	×	×	×	×	×	×	×	×	×	**15**
7	Mei et al. [17] (2021)	Ns vs. PD	×	√	√	√	√	√	√	×	√	×	×	**78**
8	Alzubaidi et al. [4] (2021)	Ns vs. PD	√	√	×	√	√	√	√	×	×	×	×	**108**
9	Proposed	Ns vs. PD	√	√	√	√	√	√	√	√	√	√	√	**105**

B1: Citation, B2: Objective, B3: Demographic discussion, B4: Inclusive and Exclusive criteria, B5: Data Extraction, B6: Model Evaluation, B7: PRISMA Model, B8: Attribute Analysis, B9: Input parameter analysis, B10: Benchmarking analysis, B11: Cross-validation, B12: Bias studies, B13: Scientific validation, B14: Reference studies, “√” article includes particular benchmark, “×” article does not includes particular benchmark.

## Data Availability

No data availability.

## Abbreviations

SN	Abb*	Definition	SN	Abb*	Definition
1	AI	Artificial intelligence	25	HMI	Human-machine interface
2	AA	Attribute Analysis	26	IEC	Inclusive and Exclusive criteria
3	AUC	Area under curve	27	IPA	Input parameter analysis
4	ACC	Accuracy	28	InParm	Input parameter
5	ARC	Architecture	29	MAE	Mean absolute error
6	BA	Benchmarking analysis	30	ME	Model Evaluation
7	BN	Batch normalization	31	ML	Machine learning
8	BS	Bias studies	32	NPV	Negative predictive value
9	BI	Brain interface	33	MSE	Mean square error
10	OB	Objective	34	PCA	Principal component analysis
11	CV	Cross-validation	35	PE	Performance evaluation indicator
12	CT	Classifier type	36	RoB	Risk of bias
13	CNN	Convolution neural network	37	RNN	Recurrent neural network
14	CONV	Convolution	38	RF	Random forest
15	CVD	Cardiovascular disease	39	SEN	Sensitivity
16	DL	Deep learning	40	SV	Scientific validation
17	DT	Decision tree	41	SDL	Solo deep learning
18	DE	Data Extraction	42	SPE	Specificity
19	DD	Demographic discussion	43	SVM	Support vector machine
20	DS	Data set	44	P	Precision
21	DSE	Dataset Size	45	R	Recall
22	ET	Ethnicity	46	RS	Reference studies
23	EEG	Electroencephalography	47	PD	Parkinson’s Disease
24	HAR	Human activity recognition	48	Abb*	Abbreviations

## Appendix A

Every study graded correlated with the attributes; a total of 30 attributes were considered for evaluation purposes and clustered into six sections. The interpret grading is applied to every cluster, according to the explanation, and every cluster was evaluated.

**Table A1:** Grading sheet.

Cluster Type	A#	Name of Attributes	#A/C	Grading Scheme (G*)
**Cluster 1** (Publications Details)	A1	Citation	3	5 (G = 3); 3 (G < 3); 1 (G < 2)
	A2	Year of Publication		
	A3	Impact Factor		
**Cluster 2** (Objective)	A4	Objective	4	5 (G = 4); 4 (G < 3); 3 (G < 2); 1 (G = 1)
	A5	Dataset Used		
	A6	Dataset Size		
	A7	Diagnosis Method		
**Cluster 3** (AI Architecture)	A8	AI Type	7	5 (G = 7); 4 (G < 5); 3 (G < 4); 2 (G< 3); 1 (G < 2); 0 (G = 1);
	A9	Architecture Used		
	A10	Internal Layers Used		
	A11	Type of Classifiers		
	A12	Data Pre-Processing		
	A13	Feature Extraction		
	A14	Activation Function		
**Cluster 4** (Optimization)	A15	Learning/Optimization Algorithm	3	5 (G = 3); 4 (G < 2); 2 (G = 1); 0 (G = 0)
	A16	Evaluation Metrics Used for Classification		
	A17	Comparison With		
**Cluster 5** (Performance)	A18	Accuracy	7	5 (G = 7); 4 (G < 5); 3 (G < 4); 2 (G < 3); 0 (G = 1)
	A19	Sensitivity		
	A20	Specificity		
	A21	AUC		
	A22	MCC		
	A23	NPV		
	A24	F1		
**Cluster 6** Clinical Evaluation and Benchmarking	A25	Demographic	6	5 (G = 6); 4 (G < 5); 3 (G < 4); 2 (G < 3); 0 (G = 1);
	A26	Age		
	A27	Ethnicity		
	A28	Validation		
	A29	Seen Vs. Unseen		
	A30	Treatment		

A#: Attribute Number, A/C: # of attributes per cluster; G*: # of Qualifying attributes per cluster.

## Appendix B

Attributes studies of 11 articles for the early detection of PD by using AI. To interpret the results of every study, a systematic approach of attributes analysis and performance indication was completed.

**Table A2:** Studies vs. Attributes.

SN	A0	A1	A2	A3	A4	A5	A6	A7	A8
	Citations	DS	DSE	ET	Age (yrs)	InPram	Arch	CT	ACC (%)
1	Alzubaidi et al. [30] (2021)	ACM	1011	Asian	60	Tremor	HDL	SVM, CNN	87.9
2	Ahmed et al. [31] (2021)	UCI	104	Asian	60	Voice	ML	RNN	95.8
3	Mei et al. [17] (2021)	PubMed, IEEE	209	Europe	60	Voice	ML	SVM	83.07
4	Singamaneni et al. [22] (2021)	UCI	410	Asian	50	Voice	ML	LDR	94.86
5	Jaichandran et al. [20] (2020)	UCI	129	Asian	60	Voice	ML	SVM, ET, *K*-Mean	78.34
6	Anitha et al. [6] (2020)	UCI	467	Asian	50	Voice	ML	CNN	90.21
7	Maitín et al. [15] (2020)	ACM, IEEE	780	America	60	EEG	ML	SVM	62.99
8	Poorjam et al. [13] (2019)	PPMI	24	Australia	50	Voice	HDL	iHMM	96.00
9	Aseer et al. [1] (2019)	MNIST	255	Asian	65	Handwriting	SDL	CNN	98.28
10	Naghsh et al. [35] (2019)	ELAB	20	Asian	50	EEG	SDL	ICA, SVM, *K*-Mean	97.38
11	Wang et al. [6] (2017)	PPMI	584	Asian	50	Biomarker	HDL	CNN, SVM, RF, NB, BT	96.12

DS: Dataset, DSE: Dataset Size, ET: Ethnicity, InParm: Input Parameter, ARC: Architecture, CT: Classifier Type, ACC: Accuracy.

## Appendix C

Performance parameters of 12 studies aligned with the type of input and AI architectures. The AI-based detection of the PD can be achieved by using symptoms as an input parameter for the algorithm. The majority of the studies explain voice as an input parameter for the diagnosis of PD. Tremor, EEG, sketch, and biomarker (chemical) data are also important input parameters to detect the PD.

**Table A3:** Twelve studies showing input data type, AI architecture, and performance parameters.

Attributes (Left to Right)	A0	A1	A2	A3	A4	A5	A6	A7
Citations	IP	AI	ACC	SEN	SPE	AUC	MCC	F1
Alzubaidi et al. [30] (2021)	Tremor	HDL	87.9	-	-	-	89.34	1.17
Ahmed et al. [31] (2021)	Voice	ML	95.8	90.24	92.3	-	92.03	96
Mei et al. [17] (2021)	Voice	ML	83.07	-	-	0.91	-	-
Singamaneni et al. [22] (2021)	Voice	ML	94.86	-	-	-	-	-
Jaichandran et al. [20] (2020)	Voice	ML	78.34	-	-	-	-	-
Anitha et al. [6] (2020)	Voice	ML	90.21	1.8	4.39	2.49		1.17
Maitín et al. [15] (2020)	EEG	ML	62.99	0.9067	0.981	-	-	-
Poorjam et al. [13] (2019)	Voice	HDL	96.00	-	-	-	-	-
Aseer et al. [1] (2019)	Handwriting	SDL	98.28	-	-	-	-	
Naghsh et al. [35] (2019)	EEG	SDL	97.38	0.9891	0.987	-	-	-
Wang et al. [6] (2017)	Biomarker	HDL	96.12	-	-	-	-	-

IP: Input Parameter, ACC: Accuracy, SEN: Sensitivity, SPE: Specificity, MCC: Matthew’s correlation coefficient.
